# The Complexities of Epidemiology and Prevention of Gastrointestinal Cancers

**DOI:** 10.3390/ijms131012556

**Published:** 2012-10-01

**Authors:** Saba Haq, Shadan Ali, Ramzi Mohammad, Fazlul H. Sarkar

**Affiliations:** 1 Department of Pathology, Karmanos Cancer Institute, Wayne State University School of Medicine, Detroit, MI 48201, USA; E-Mail: shaq.87@gmail.com; 2 Department of Oncology, Karmanos Cancer Institute, Wayne State University School of Medicine, Detroit, MI 48201, USA; E-Mails: alis@karmanos.org (S.A.); mohammar@karmanos.org (R.M.)

**Keywords:** cancer epidemiology, prevention, pancreatic cancer, colon cancer

## Abstract

Cancer epidemiology and prevention is one of the most well studied fields today. The more we can understand about the incidence and pathogenesis of this disease, the better we will be able to prevent it. Effective prevention strategies can decrease the mortality rate of cancer significantly; this is why it is important to delineate the underlying causes. It has been well recognized that genetic mutations, sporadic or hereditary, may lead to increased chance of tumorigenesis. Detecting genetic mutations can lead to the identification of high-risk individuals with hereditary cancer syndromes, which may assist in devising prevention strategies. Further, environmental factors are known to play important roles in epidemiology and suggest prevention tools that could be implemented to reduce cancer incidence and subsequent cancer-associated morbidity and mortality. Chemoprevention has been tried in colon cancer and is finding new advancements in other carcinomas as well. Out of many environmental cancer preventive agents, the most notable developments are the identification of the role of vitamins E, vitamin D and folic acid. Increased consumption of these vitamins has shown to be inversely correlated with cancer risk. This review will highlight important aspects of cancer epidemiology in the most aggressive carcinomas of the gastrointestinal system focusing on colorectal adenocarcinoma and pancreatic adenocarcinoma. Additionally, some of the well-known and evolving aspects of epidemiology of colorectal and pancreatic cancer along with current and new prevention strategies will also be reviewed.

## 1. Introduction

When discussing the preventive aspects of cancer, keeping cancer epidemiology in perspective is crucial. Epidemiology tells us the, who, what, how, and when regarding a disease. Ultimately, it will serve as an aid to show us where to concentrate our prevention efforts in the most effective manner. In cancer, many of these aggressive carcinomas are detected in a stage where it is too late. For this reason, it is imperative to use epidemiological understanding to develop prevention programs aimed at reducing the high mortality rate. Colon and pancreatic carcinomas are some of the most aggressive gastrointestinal (GI) carcinomas known today. Routine screening has been recommended by the United States Preventative Services Task Force (USPSTF) for colon carcinoma, but not pancreatic carcinoma. However, the fact that the mortality rate remains relatively high from these cancers suggests that screening may not be the only effective tool, but prevention may be the key for decreasing mortality from these carcinomas.

Hereditary cancer syndromes as well as sporadic genetic mutations are well known causes of cancer [1]. Genetic mutations can make a patient more susceptible to cancer especially when combined with environmental factors such as the effect of smoking on pancreatic cancer [2]. Recognizing genetic mutations would identify high-risk patients who could be regularly screened. This would aid in decreasing mortality rate as it would identify a higher number of cancers at an earlier stage than routine screening [3].

Environmental influences have been shown in carcinogenesis and prevention of carcinomas in general. An important role played by fat-soluble vitamins has been elucidated, namely with vitamins D and E and folic acid. Recent studies have shown the protective role of isoflavone in carcinogenesis [4]. Among all these vitamins, the main effect observed was due to the antioxidant property of these elements. It may be the imbalance of low antioxidants and high level of free radicals, which may enhance the process of carcinogenesis [5]. Researchers have observed that a cumulative effect between all these vitamins may account for protective effect in cancer [6,7]. The interaction of these natural elements with anti-apoptotic pathways also plays an important role [4]. Smoking cessation has long been included in the prevention of most cancers, especially lung cancer as well other diseases and continues to be strongly encouraged [8,9]. Links to obesity, alcohol and chronic inflammation have also been identified in the pathogenesis of carcinoma [10–12].

Chemoprevention is promising in the realm of cancer prevention. It has been shown to reduce the risk of development of carcinoma in highly susceptible individuals such as those with known genetic mutations or high level of risk factors. A negative factor in using chemo- protective agents is the side effects. Side effects must be taken into account and one must weigh the benefits over the risks of taking that drug. For this reason, chemoprevention is usually not administered in the general low risk population [13]. The role of COX-2 inhibitors in colon cancer prevention and metformin in pancreatic cancer as chemopreventive agents will be discussed below in this chapter.

In this chapter, we will discuss the epidemiology and prevention of aggressive gastrointestinal (GI) cancers with a high mortality rate: colon and pancreatic cancer. The existing knowledge and evolving new information of the epidemiology of each cancer will be discussed. Strategies which are currently being implemented along with newer and more effective approaches are still being investigated for the prevention of these carcinomas will be discussed in each section.

## 2. Colon Cancer

Colorectal carcinoma (CRC) is one of the most common causes of mortality in the United States and worldwide. Around 600,000 deaths are attributed to this cancer including sporadic and hereditary forms [14]. Due to its aggressive nature, better screening and prevention methods are being tested in order to reduce the burden of this disease. There are many causes of colon cancer involving both genetic and environmental influences and these are discussed below.

## 3. Hereditary Colon Cancer

It has been shown that 30% of colorectal carcinoma cases are part of an inherited form of cancer [15], and the common hereditary syndromes consist of Lynch syndrome and familial adenomatous polyposis, which account for the majority of hereditary cases. Patients with lynch syndrome have a high risk of developing not only colon cancer but other cancers as well especially endometrial cancer. A person with lynch syndrome has a lifetime risk of 50%–80% of developing colorectal carcinoma. It appears that they acquire the carcinoma at a younger age than sporadic forms. Analysis of the tumors shows that they are more commonly located in the proximal colon and are poorly differentiated. The genetic mutation in lynch syndrome is a germ line mutation of DNA mismatch repair genes, resulting in microsatellite instability. The genes involved include hMSH2, hMLH1, hMSH6, and hPMS2, and the full names of the genes are included in Table 1. A defect in the DNA repair genes results in genomic instability with a higher risk of mutations. The most common mutated gene accounting for 90% of lynch syndrome cases is hPMS2. Suggested approach for cancer prevention in lynch syndrome is focused on a detailed family history and assessment of risk factors. The Amsterdam criteria and Bethesda guidelines have been made for that purpose. Patients who are identified as high-risk candidates should be screened for mutations especially for hPMS2 mutations. The mutations can also be identified via immunohistochemistry analysis which could find proteins for multiple mutations in mismatch repair genes [15]. Another approach being used recently is to test every patient for mismatch repair mutations, which has led to identification of more cancer cases. However, it has been shown that the Bethesda guidelines missed almost 28% of Lynch syndrome cases. Therefore, it has been suggested that patients identified with Lynch syndrome be screened at intervals of 1–2 years starting at age 20–25 [15]. By reducing the interval between screenings, mortality rate would likely to be decreased due to identification of patients with early stage tumors. One must also keep in mind that Lynch syndrome poses a risk for endometrial carcinoma as well, and thus surveillance must be implemented for better management of patients [15].

Familial Adenomatous Polyposis (FAP) is a premalignant condition which poses almost 100% risk of developing colorectal carcinoma. It develops at a younger age than other hereditary syndromes. The diagnosis is made when there are at least 100 colonic polyps but there may be thousands of polyps. The genetic mutation responsible for FAP is a mutated *APC* gene, which encodes a tumor suppressor in the WNT pathway. Mutations of the *APC* gene can also be sporadic and can account for 25% of FAP cases. Mutations in the *APC* gene can also be associated with other syndromes such as attenuated FAP, Gardner syndrome and osteomas [15]. It has been recommended that high-risk patients should be screened every 1–2 years starting at a young age of 10–12 years [15]. Ultimately an elective procto-colectomy is indicated once FAP is identified.

## 4. Role of Inflammatory Bowel Disease in CRC

There has been a good relationship between inflammatory bowel disease (IBD) and colorectal cancer as demonstrated in mouse models of colon adenocarcinoma. It has also been shown that patients with IBD are at a 2–5 times more likely to be diagnosed with colorectal cancer than the general population [14]. Chronic inflammation of the bowel is associated with increased levels of pro-inflammatory cytokines and the increased signaling, which plays important roles in the development of carcinoma [11]. There are different mechanisms that may be responsible for the development of carcinoma in IBD patients, one being the production of reactive oxygen species (ROS). ROS can induce damage to DNA which predisposed to gene mutations, and therefore can contribute to carcinogenesis [11]. Some of the pathways involved in ROS induced carcinogenesis includes MAP kinase and phosphoinositide 3-kinase (PI3K) pathways that are induced during the development of cancer (carcinogenesis) and promotes cell survival [16–18]. Besides DNA damage, ROS can activate oncogenes and inactivate tumor suppressor genes which further elucidates the role of IBD in CRC [19]. It has been demonstrated that these oncogenes itself promote the formation of more ROS as well, and thus the activation of ROS and oncogenes creates a vicious cycle during the development of cancer [17]. Pro-inflammatory cytokines that cause inflammation and are associated with tumorigenesis include IL-1, IL-6 and TNF-α. These cytokines are involved in cell signaling via the NF-kappaB (NF-κB) pathway, a common pathway critical for the development of tumor in many organs [11].

Although defects in the mismatch repair (MMR) genes have been discussed as hereditary, sporadic defects can also be found which is typically caused by hypermethylation or by deregulation of microRNAs (miRNAs), resulting in loss of MMR activity. It has been reported that methylation occurs at a higher rate in those tissues which are exposed to inflammation. It has been shown that DNA methylation is more frequent and more common in non-neoplastic cells in the mucosa from ulcerative colitis patients with colon cancer as opposed to those with non-neoplastic mucosa from ulcerative colitis without cancer. Some of the commonly methylated genes include RUNX2 and MINT1. This data suggests that these findings can help for early detection of dysplasia and also in the identification of patients at higher risk.

## 5. Chemoprevention in Colon Cancer

Recent studies have shown that there may be a role of COX-2 inhibitors in the prevention of colon cancer. Studies have demonstrated a high level of COX-2 in patients with CRC; thereby leading researchers have tested the effect of an inhibitor on cancer patients. A study has shown that using aspirin (COX-1 and COX-2 inhibitor) in patients with Familial Adenomatous Polyposis had a 21% reduction on the risk of adenoma recurrence and a possible reduction in the polyp size; however, a reduction in the number of polyps was not significant [20]. When aspirin was tested in the general population, a 26% reduction of CRC incidence was observed when patients were followed up for 23 years. A 34% decrease in adenoma recurrence was also demonstrated in patients with a history of adenomas [20]. These results suggest that the uses of non-steroidal anti-inflammatory drugs (NSAIDs) like aspirin and celecoxib (COX-2 inhibitor) have profound effect on the development and progression of an adenoma or CRC although there are some side effects for long-term use of COX-2 inhibitors. Among many side effects, renal and gastrointestinal symptoms such as nausea vomiting, diarrhea and increased risk of bleeding are more common for short-term use [21]. For this reason, COX-2 inhibitors may be considered in those with high-risk adenomas who may gain higher benefit to risk ratio of using these drugs. Despite its side effects, COX-2 inhibitors have a high potential to be a novel drug for chemoprevention especially because it is cost effective and accessible to everyone, especially aspirin, which targets both COX-1 and COX-2 [21].

Mesalazine, a drug commonly used to reduce the inflammation of ulcerative colitis and crohns disease may be a favorable drug for the prevention of colon carcinoma [22,23]. Mesalazine is an agent which may exerts apoptotic effects on colon cancer cells and may also interfere with the cell cycle of neoplastic cells; however, its role as a chemopreventive agent in inflammatory bowel induced carcinoma is still being investigated [22].

## 6. The Role of Nutritional Agents in Colon Cancer

Nutritional factors are increasingly found to play important roles in the prevention of carcinomas, and delaying the progression of cancerous lesions. Folic acid is a water-soluble vitamin that has been shown to decrease the incidence, size, and multiplicity of adenocarcinoma in animal models. Folic acid supplementation has also been shown to have more effect in pre-adenoma stage as opposed to the post-adenoma stage [24]. Folic acid can affect DNA methylation and DNA synthesis by providing methyl groups. These are the same steps in which mutations can lead to cancer progression [24]. A recent study has shown that Folic acid intake 12–16 years before diagnosis was inversely related with the development of CRC [24]. On the other hand it was demonstrated that folate deficiency can enhance the development of neoplastic lesions compared to those who had an intake of 8 mg/kg of folic acid. A healthier diet rich in folic acid could be helpful in the prevention of colon cancer and may also affect other carcinomas as well. Other dietary factors that can help protect the colon include high fiber diet, low red meat intake, and increased physical activity [25–27].

Besides a healthy diet, a healthy lifestyle and low body mass index (BMI) could help decrease the risk of CRC. Microsatellite instability has been discussed above as a cause for hereditary cancer. Here we discuss that a high BMI is associated with the development and progression of carcinoma in those limited to microsatellite stability (MSS) [28]. For every BMI increase of 5 kg/m^2^, the risk of carcinoma was increased (the odds ratio of 1.38 with a 95% confidence interval 1.24–1.54) [28]. The reason that obesity carries may have higher risk of colon cancer is because of increased activity of fatty acid synthase (FASN) in the tumor. Obesity may also reduce survival of patients, most likely due to interaction between the fatty acid synthase and adipose tissues. This relationship has yet to be confirmed and continues to be studied. However one must always counsel patients for the need to keep a BMI within normal range in order to decrease the risk of developing CRC, especially in high-risk patients with multiple risk factors.

## 7. Current Screening Recommendations in Colon Cancer

According to the United States Preventive Services Task Force (USPSTF), patients should be screened with Fecal Occult Blood Testing (FOBT), sigmoidoscopy, or colonoscopy in adults beginning at the age of 50 till age 75. The intervals between testing may vary depending on the test results. Annual screening for FOBT is recommended. Sigmoidoscopy and FOBT are recommended at five-year intervals and colonoscopy is recommended at 10-year intervals for subjects without any evidence of polyps or benign adenoma. High-risk individuals, especially those with hereditary cancer syndromes, have different screening intervals when compared to the general population and it is presented in Table 2. It has been shown that individuals with a higher risk of cancer require shorter interval period between screening in order to identify new lesions, which could easily be removed and pathologically examined for assessing the exact risk [29]. It has been noted that patients identified with FAP who have the highest risk of carcinoma should be tested at the earliest age [30].

According to the USPSTF, newer screening strategies such as CT colonography may be helpful in those who refuse screening by colonoscopy. It is less invasive and does not require a bowl preparation before the test. Fecal DNA testing is a promising, highly specific test which continues to evolve. The USPSTF has stated that as of now there is insufficient evidence to perform these tests as a regular practice.

## 8. Pancreatic Cancer

Pancreatic cancer is the fourth leading cause of cancer worldwide accounting for 36,800 deaths yearly in the United States. Pancreatic cancer is usually diagnosed when it is already in a late stage, and for this reason the mortality rate is high for this cancer [31]. Although there are no current screening recommendations by the USPSTF, primary prevention could be through dietary and environmental changes, but genetic screening has been suggested to identify high-risk subjects as discussed below [32].

## 9. Hereditary Pancreatic Cancer

It has been demonstrated that hereditary pancreatic cancer is responsible for 10% of all pancreatic carcinomas [31]. Hereditary pancreatic carcinoma is commonly inherited as a syndrome in which one mutation leads to susceptibility to multiple carcinomas. Examples of such syndromes are Lynch syndrome with the mutation of the mismatch repair genes. These tumors tend to be poorly differentiated and have prominent lymphocytic infiltration [31].

A significant association between BRCA2 mutation and pancreatic cancer has also been demonstrated. It has been shown that in four out of eight carcinomas, an underlying BRCA2 mutation was identified [33]. Patients with a BRCA2 mutation are ten times more likely to develop pancreatic cancer compared with the average risk population [33]. Thus, identification of the BRCA2 gene in a patient would be a reason to screen for an associated pancreatic cancer, especially in the Ashkenazi Jewish population who have a high rate of BRCA2 mutations [34]. There are many other cancer syndromes which can increase the risk of pancreatic carcinoma. The syndromes along with their mutation are presented in Table 3. Genetic screening for these selective mutations should be done and once discovered, there should be a high suspicion for the development of pancreatic carcinoma [35], and thus these subjects must be clinically managed.

Familial pancreatic cancer has been demonstrated to occur in clusters within families and tend to occur at an earlier age than sporadic carcinoma. Also when combined with environmental factors, the patients have a lower threshold for developing carcinoma [7]. For example, a study was done to demonstrate the risk of cancer in smokers with a positive and negative history of first degree relatives with pancreatic carcinoma. The study showed that smokers with a positive family history with first degree relatives with the disease had almost twice the risk of pancreatic adenocarcinomas compared to those with a negative family history in first degree relatives [7]. Targeting these genetic mutations have the ability to prevent cancer in individuals who are at a higher risk, and thus more efficient screening can be done on those patients rather than focusing on the whole population, thereby making this method of screening cost effective [7]. Prophylactic surgery has been considered in those with hereditary cancer syndromes; however, no recommendations have been made by the USPSTF regarding pancreatic cancer [36].

## 10. Role of Smoking on Pancreatic Cancer

The role of smoking in cancer has long been elucidated, and it is a well-known risk factor especially for lung, pancreatic and colon cancer. Smokers are three times more likely to develop pancreatic carcinoma and at an earlier age than non-smokers. A combined family history and smoking status of the patient would increase the chance of carcinoma even greater, possibly due to a cumulative effect [7]. The main ingredient in cigarettes with profound carcinogenic effects is nicotine and its active metabolites, NNK and NNN, known carcinogens. The main effects of nicotine in the development of pancreatic cancer is by activating pathways altering the level of surface receptors and signaling peptides such as EGFR receptor, cAMP and ERK 1 and 2 in pancreatic cells. An increased level of osteopontin which is responsible for increased expression of VEGF mRNA has been reported. All these receptors are the same ones responsible in the process of carcinogenesis, proliferation, angiogenesis, metastasis and decreased apoptosis [37].

Smoking cessation has been recommended for years based on its implication in multiple diseases including cancer. The benefits of smoking cessation have shown immediate improvements as well as long-term improvements in patient health. More awareness should be made about the relationship between smoking and pancreatic cancer in order to reduce the related mortality.

## 11. Role of Obesity and Diabetes in Pancreatic Carcinoma

Obesity is being established as a risk factor for pancreatic cancer along with diabetes and decreased physical activity. A BMI of more than 25 kg/m^2^ was associated with an increased risk of pancreatic cancer of about 2.5 fold, and diabetes mellitus has been associated with a 2.9 fold increased risk [12]. A high BMI may cause damage to DNA through increased lipid peroxidation in the body. A high level of adipose tissue in obese individuals may be the cause of increased lipid peroxidation in these individuals. Every 5 kg/m^2^ increase in BMI was associated with an increased risk of pancreatic cancer [12,38].

One gene associated with obesity and diabetes mellitus that has significance in pancreatic carcinoma is PPARγ. The functions of PPARγ include not only lipid and glucose homeostasis but also the suppression of pro-inflammatory transcription factors like NF-κB and AP-1 [39]. PPARγ exerts its carcinogenic effects mainly by stimulating angiogenesis. Since PPARγ is also increased significantly in obese patients due to increased adipose tissue, the risk for pancreatic carcinoma is increased.

## 12. Metformin as a Chemopreventive Agent

Recent studies are beginning to demonstrate that metformin may prove to be a novel chemo preventative agent for pancreatic carcinoma. Studies have shown that the intake of metformin, an oral hypoglycemic agent, has been associated with a decreased risk of pancreatic carcinoma. In addition to its effects on blood sugar levels, metformin has been shown to inhibit proliferation of cells as well as stimulate apoptosis. Therefore, treatment of diabetes, which itself is a risk factor metformin showed anti-tumor characteristics as well. Patients treated with metformin demonstrated improved endothelial function including the expression of adhesion molecules, E-selectin and Vascular Endothelial Growth Factor (VEGF); all of which are important factors in promoting tumorigenesis [32]. A decrease in risk by 62% has been demonstrated in metformin users compared to non-users [32,40].

## 13. Alcohol and Chronic Pancreatitis in Pancreatic Carcinoma

The role of alcohol in the epidemiology of esophageal, colorectal, liver and oral cancers has been elucidated; however, the role of alcohol in pancreatic carcinoma is yet to be confirmed. The causal relationship with carcinoma may be mediated through its role in causing chronic pancreatitis [41,42]. There is a strong correlation between alcohol use and pancreatic carcinoma associated with confounding factor such as smoking. In a study done to examine the causal relationship, it was demonstrated that the incidence of pancreatic carcinoma was 46.4 per 100,000 person years among heavy drinkers compared to only 29.9 in light drinkers [41]. Patients who drink heavy amounts of liquor had the highest rate of developing carcinoma at an increased risk of 62% compared to the general population [41]. Besides the type of alcohol used, the amounts in one sitting have also been shown to be of significance. Binge drinking for example is associated with higher risk of carcinoma than regular drinking. The risk was increased to three times as much regardless of when the binge drinking occurred. However this risk was only significant when it came to men and was not statistically significant in women [10].

Many explanations have been suggested for the effect of alcohol. Subjects who are heavy drinkers tend to be deficient in folate, and because folate has been shown to be protective, and thus subjects deficient in folate may be susceptible to the development of carcinoma [43]. Heavy liquor contains nitrosamines and polycyclic aromatic hydrocarbons, which are well known carcinogens, and therefore the possible effect of alcohol metabolites could results in the production of ROS, which causes DNA damage, which appears to be in part responsible for the development of carcinoma. Current opinion for the causal relationship between alcohol and pancreatic carcinoma is due to long term inflammation, and the fibrosis inducing effect on the pancreas leading to chronic pancreatitis or even diabetes mellitus which are both pro-carcinogenic [10,41], which leads to the development of pancreatic cancer.

## 14. Role of Natural Agents in Cancer Epidemiology and Prevention

### 14.1. Fat Soluble Vitamins

Fat-soluble vitamins have shown to have important roles in epidemiology and prevention of gastrointestinal cancers. Of note, vitamin E succinate has significant biochemical effects such as inhibition of cell proliferation and induction of apoptosis in all cells including pancreatic cells. It has been suggested that one mechanism by which vitamin E exerts its anti-carcinogenic function is mediated through deregulated expression of survivin. Survivin is an inhibitor of cell apoptosis, thereby an inducer of carcinogenesis [44,45]. It has been demonstrated that inhibition of survivin enhanced apoptosis by vitamin E in pancreatic cancer cell lines [46], and interacts with signaling pathways such as transforming growth factor-beta and Fas, which are both involved in apoptotic pathways [46].

Vitamin E mainly exerts antioxidant effects in the body. Its function is to neutralize the effects of ROS which would otherwise damage human DNA. ROS causes the formation of disulfide bridges leading to the inhibition of apoptosis, which could be attenuated by vitamin E succinate [46]. Based on these findings, vitamin E succinate has been shown to function as a potential novel agent for the prevention of pancreatic carcinoma [47] especially mediated through deregulation of survivin, and thus survivin could be a target for chemotherapy, which may prove to be valuable in the prevention of cancers [46].

### 14.2. Vitamin D

Vitamin D has been shown to be useful as a chemopreventive agent in a multitude of cancers including pancreas, colon, and breast carcinomas. It has been documented that an increased intake of vitamin D in the diet could prevent around 100,000 cases of cancer alone [47]. The active form of vitamin D is calcitriol which has multiple cancer protective effects. Its role as a chemopreventive agent is still being studied; however, it does hold promise. Calcitriol is highly involved in the pathways of cell signaling and apoptosis as well as adhesion of cells and angiogenesis. It has also been shown to up-regulate tumor suppressors such as p21 and p27 [48]. With regards to the cell cycle, vitamin D, through its interactions with Cyclin D, can inhibit cells from completing the cell cycle. Also by increasing the amount of E-cadherin, vitamin D can prevent tumorigenesis by stopping the proliferation of neoplastic cells. Calcitriol can decrease angiogenesis by decreasing vascular endothelial growth factor (VEGF), which is necessary for the proliferation of blood vessels [48]. It has been shown that there is an increase of 1 α hydroxylase, the activator of vitamin D, in pancreatic cancer cells which may prove why vitamin D may be a novel agent in the prevention of pancreatic carcinoma [48]; however, further mechanistic *in vitro* and *in vivo* studies are warranted. Based on its effects on the cell cycle and angiogenesis, vitamin D intake should be increased in any patient in the general population as it is cost effective and its benefits to the body are substantial [49].

## 15. Role of Natural Anti-Cancer Agents

Emerging developments in the field of cancer prevention research are now being appreciated due to the potential role of natural agents both in the prevention of cancer and in the treatment of multiple malignancies by using these agents alone or in combination with conventional therapeutics.

### 15.1. Ciclopirox (CPX)

An important agent known as ciclopirox (CPX) which has been used as an anti fungal agent for years is now getting attention as an anti-cancer agent. It is main mechanism is mediated through its ability to prevent the cell cycle from progression to the S phase [50]. CPX has also been shown to act through p53 independent mechanisms. Further studies have shown that CPX could inhibit proteins such as survivin and Bcl-XL which are anti-apoptotic proteins [50].

### 15.2. Cryptotanshinone (CPT)

Another agent known as cryptotanshinone (CPT), a natural agent that have been used in oriental medicine has shown similar effects to that of CPX. It has been demonstrated that CPT could arrest the cell cycle in the G1/G0 phase [51]. The arrest of the cell cycle was in part related to the inhibition of cyclin D, a regulator of the cell cycle, as well as by decreased phosphorylation of Rb (Retinoblastoma) protein [51]. In addition, another main pathway that have been shown to be involved in this process was the mTOR pathway, which is a mediator of activation of cyclin D [51].

### 15.3. Curcumin

Curcumin, found in turmeric is a common agent used in cooking foods in the south-east Asian population, and has been demonstrated to show antitumor activity. Curcumin has been shown to function as a novel anticancer agent because it is very safe to humans and has only limited toxicity [52,53]. There are multiple mechanisms by which curcumin elicits its effects on cancer cells. Curcumin inhibits the activity of the enzyme cytochrome p-450, thereby increasing the levels of glutathione-*s*-transferase activity, having an anti-oxidant effect on the body [53]. It has been shown that curcumin could rapidly induce MAPKs including Erk1/2 and c-Jun *N*-terminal kinase (JNK) [54]. It has also has been shown to possess similar effect as CPX by acting on the cell cycle and causing cell cycle arrest of cancer cells leading to the inhibition of cell proliferation. An interesting effect of curcumin specific to colorectal cancer is that curcumin inhibits growth of colonic cells which have defective DNA repair capacity due to lack of functional mismatch repair enzymes [53]. This makes curcumin a novel agent to be used in colorectal cancer and other cancers as well.

### 15.4. Rapamycin

Rapamycin is another FDA-approved antibiotic and immunosuppressant, which is currently being tested in phase II and phase III clinical trials in cancer patients for its anti-tumor activity. It inhibits mTOR signaling pathway. According to Liu *et al.,* rapamycin inhibits insulin-like growth factor 1 (IGF-1) stimulated cell motility by activating serine/threonine protein phosphatase 2A and phosphorylation of Erk 1/2 [55]. Recent studies from our laboratory have demonstrated that rapamycin inhibits cell proliferation of PDGF-D over-expressing PC3 cells by suppressing the activity of the mTOR signaling pathway [56].

### 15.5. Isoflavone

Isoflavone are gaining a great deal of attention because of its role as a natural agent responsible for the preventive and therapeutic effects on cancer cells. Isoflavone are agents which can be found in soybeans, lentils, peas and other beans. However, the isoflavone found in soybeans have been extensively studied [4,44,57–61]. Isoflavone, more specifically genistein has shown profound effects on the body comparable to the effect that fat-soluble vitamins have on the body. Interestingly, genistein has been shown to function as an antioxidant, which protects cells from ROS and prevent DNA damage from free radicals [4]. Isoflavone has also shown to have a significant effect on cell cycle and as an inducer of apoptosis mainly via inhibition of NF-κB [62–64]. Other pathways that have been implicated in mediating the biological activity of isoflavone are mainly the Akt, Wnt, MAPK, and Notch pathways, all of which are involved in anti-apoptotic mechanisms [4,44,59]. In this regard, isoflavone in the diet are very important and should be considered in all patients and the patients should also be counseled on the beneficial effects and importance of this natural agent along with the other vitamins [65]. A proper diet filled with these vitamins and minerals and excluding harmful environmental factors such as smoking and alcohol should be able to prevent some cancers while at the same time they are being resourceful and cost effective.

## 16. Conclusion

Based on our discussion on the subject of this chapter, we can conclude that understanding the mechanisms behind the development of carcinoma is crucial in order to cultivate methods to either intervene or prevent this human tragedy. Genetic elements have proven to play important roles in both hereditary and sporadic cancers. Both, colorectal and pancreatic cancers have genetic elements which if discovered early, could help to decrease the incidence of cancer. The main methods that are currently being used to identify carcinoma at an early stage are through obtaining thorough family history, identification of risk factors, and ultimately performing genetic testing to screen for these mutated genes. Moreover, inflammatory conditions have the propensity to induce oncogenes and inactivate tumor suppressor genes as well as cause DNA damage, and therefore plays important role in carcinogenesis [66]. Inflammatory bowel disease model provide an opportunity for chemoprevention to reduce the effects of inflammation by using COX-2 inhibitors and also the use of Mesalazine [20,22,65]. Moreover, metformin is proving to be a promising chemopreventive agent especially in diabetes induced pancreatic adenocarcinoma; however, chronic pancreatitis maybe more benefited from preventive strategies such as counseling to decrease consumption of alcohol use in the patient. Lifestyle factors are also proving to be imperative in terms of prevention in gastrointestinal carcinomas. It is of utmost importance to counsel patients in the clinical setting about the carcinogenic effects of smoking and alcohol intake. A diet rich in vitamin E and D as well as folic acid and isoflavone can have profound beneficial effects in terms of anti-carcinogenic activity. The type of diet can help to maintain normal BMI, thereby reducing risks of both colorectal and pancreatic carcinoma. In conclusion, we have provided deeper insights into the current trends in epidemiological sciences specifically related to detection and prevention of gastric-carcinomas. From the above comprehensive set of information, it is clear that the alterations in diet and lifestyle could make a difference either in the maintenance of normal physiology or the development of neoplastic disease.

## Figures and Tables

**Table 1:** Important genes in colon and pancreatic cancer.

Abbreviated Genes	Full name of Genes
hMSH2	human mutS homolog 2
hMLH1	human mutL homolog 1
hMSH6	human mutS homolog 6
hPMS2	postmeiotic segregation increased 2
APC	adenomatous polyposis coli
RUNX2	Runt-related transcription factor 2
MINT1	munc-18 interacting protein
BRCA1/2	Breast cancer 1/2
PPARγ	Peroxisome proliferator-activated receptor gamma

**Table 2:** Colon cancer screening.

	Screening Initiation (age)	Screening Interval
General Population	50	5–10 years
Family History of Colon Cancer	10 years before age family member was diagnosed or 50, whichever comes earlier	
Lynch Syndrome	20–25	1–2 years
Familial Adenomatous Polyposis	10–12	1–2 years
Patients with Inflammatory Bowel Disease	Begin after 8 years of disease	Yearly

**Table 3:** Inherited pancreatic cancer syndromes.

Inherited Syndrome	Mutated Gene
Lynch Syndrome	Mismatch Repair Genes (MMR): MLH1, MSH2, MSH6 and PMS2
Peutz-Jeghers Syndrome	STK11/LKB1
Hereditary Breast Cancer	BRCA1/BRCA2
Familial Atypical Multiple Mole Melanoma	CDKN2A mutation
